# The Add-On Effect of *Lactobacillus plantarum* PS128 in Patients With Parkinson's Disease: A Pilot Study

**DOI:** 10.3389/fnut.2021.650053

**Published:** 2021-06-30

**Authors:** Chin-Song Lu, Hsiu-Chen Chang, Yi-Hsin Weng, Chiung-Chu Chen, Yi-Shan Kuo, Ying-Chieh Tsai

**Affiliations:** ^1^Professor Lu Neurological Clinic, Taoyuan, Taiwan; ^2^Division of Movement Disorders, Department of Neurology, Chang Gung Memorial Hospital at Linkou, Taoyuan, Taiwan; ^3^School of Medicine, College of Medicine, Chang Gung University, Taoyuan, Taiwan; ^4^Neuroscience Research Center, Chang Gung Memorial Hospital at Linkou, Taoyuan, Taiwan; ^5^Institute of Biochemistry and Molecular Biology, National Yang Ming Chiao Tung University, Taipei, Taiwan

**Keywords:** *Lactobacillus plantarum*, PS128, Parkinson's disease, probiotics, psychobiotics

## Abstract

**Background:** *Lactobacillus plantarum* PS128 (PS128) is a specific probiotic, known as a psychobiotic, which has been demonstrated to alleviate motor deficits and inhibit neurodegenerative processes in Parkinson's disease (PD)-model mice. We hypothesize that it may also be beneficial to patients with PD based on the possible mechanism via the microbiome-gut-brain axis.

**Methods:** This is an open-label, single-arm, baseline-controlled trial. The eligible participants were scheduled to take 60 billion colony-forming units of PS128 once per night for 12 weeks. Clinical assessments were conducted using the Unified Parkinson's Disease Rating Scale (UPDRS), modified Hoehn and Yahr scale, and change in patient “ON-OFF” diary recording as primary outcome measures. The non-motor symptoms questionnaire, Beck depression inventory-II, patient assessment of constipation symptom, 39-item Parkinson's Disease Questionnaire (PDQ-39), and Patient Global Impression of Change (PGI-C) were assessed as secondary outcome measures.

**Results:** Twenty-five eligible patients (32% women) completed the study. The mean age was 61.84 ± 5.74 years (range, 52–72), mean disease duration was 10.12 ± 2.3 years (range, 5–14), and levodopa equivalent daily dosage was 1063.4 ± 209.5 mg/daily (range, 675–1,560). All patients remained on the same dosage of anti-parkinsonian and other drugs throughout the study. After 12 weeks of PS128 supplementation, the UPDRS motor scores improved significantly in both the OFF and ON states (*p* = 0.004 and *p* = 0.007, respectively). In addition, PS128 intervention significantly improved the duration of the ON period and OFF period as well as PDQ-39 values. However, no obvious effect of PS128 on non-motor symptoms of patients with PD was observed. Notably, the PGI-C scores improved in 17 patients (68%). PS128 intervention was also found to significantly reduce plasma myeloperoxidase and urine creatinine levels.

**Conclusion:** The present study demonstrated that PS128 supplementation for 12 weeks with constant anti-parkinsonian medication improved the UPDRS motor score and quality of life of PD patients. We suggest that PS128 could serve as a therapeutic adjuvant for the treatment of PD. In the future, placebo-controlled studies are needed to further support the efficacy of PS128 supplementation.

**Clinical Trial Registration:** https://clinicaltrials.gov/, identifier: NCT04389762.

## Introduction

Parkinson's disease (PD) is a neurodegenerative disorder associated with motor and non-motor symptoms (1). The prevalence of PD increases with age, and PD affects 1% of the population over 60 years (2). Dopaminergic medications are effective treatments for PD patients. However, there are several side effects of dopaminergic drugs such as motor fluctuations, dyskinesia, and other psychological behaviors (3). The key pathological features of PD include a selective loss of dopamine-producing neurons in the substantia nigra, a region in the midbrain, and the presence of Lewy bodies, which consist primarily of misfolded α-synuclein (4). For decades, scientists focused on the substantia nigra to study how the disease process actually begins; however, accumulating evidence suggests that in some patients, PD originates in the gut (5). Clinically, the non-motor symptoms, including gastrointestinal manifestations, often appear years before the motor symptoms and aggregates of α-synuclein have been found in enteric nerves of patients with PD (6). Enteroendocrine cells (EECs), which are sensory epithelial cells of the gut, are able to express α-synuclein and synapse with enteric nerves (7); patients undergoing vagotomy, a surgical procedure that removes all or part of the vagus nerve, have a reduced risk of developing PD (8). Furthermore, gut dysbiosis has been suggested to be a contributing factor to the pathophysiology of PD (1, 9). These findings also indicate possible uses of food-based treatments, including probiotics, for PD (10).

Probiotics are defined as live microorganisms, which when administered in adequate amounts confer a health benefit on the host (11). Specific probiotic strains, also known as psychobiotics, have been shown to confer beneficial effects on the brain via the microbiome-gut-brain axis (12, 13). Previous studies have reported a novel psychobiotic strain, *Lactobacillus plantarum* PS128 (PS128), which ameliorated abnormal behaviors and regulates both dopaminergic and serotonergic signaling in the brain of mice (14, 15). Oral administration of PS128 also modulated peripheral serotonin levels in rats (16), suggesting an interaction between PS128 and serotonin-producing EECs, which account for ~90% of the body's serotonin levels (17). Furthermore, PS128 ingestion alleviated motor deficits, nigrostriatal dopaminergic neuronal cell death, and striatal dopamine reduction in a PD mouse model (18), thus providing an insight into the use of PS128 as a potential treatment or add-on treatment alternative for patients with PD.

In previous clinical studies, PS128 has been reported to ameliorate behaviors in children with autism spectrum disorder (19) and improve physiological adaptation and performance in triathletes (20, 21). In this study, we aimed to investigate the add-on effect of PS128 supplementation for 12 weeks in patients with PD with an OFF period (the medication is not working well, and symptoms of PD have temporarily reappeared) longer than 3 h a day. Motor and non-motor symptoms, self-reported health status and quality of life, and metabolic parameters in the blood and urine of the patients were measured before and after PS128 intervention and were compared to each other.

## Methods

### Participants and Study Design

**Figure 1:**
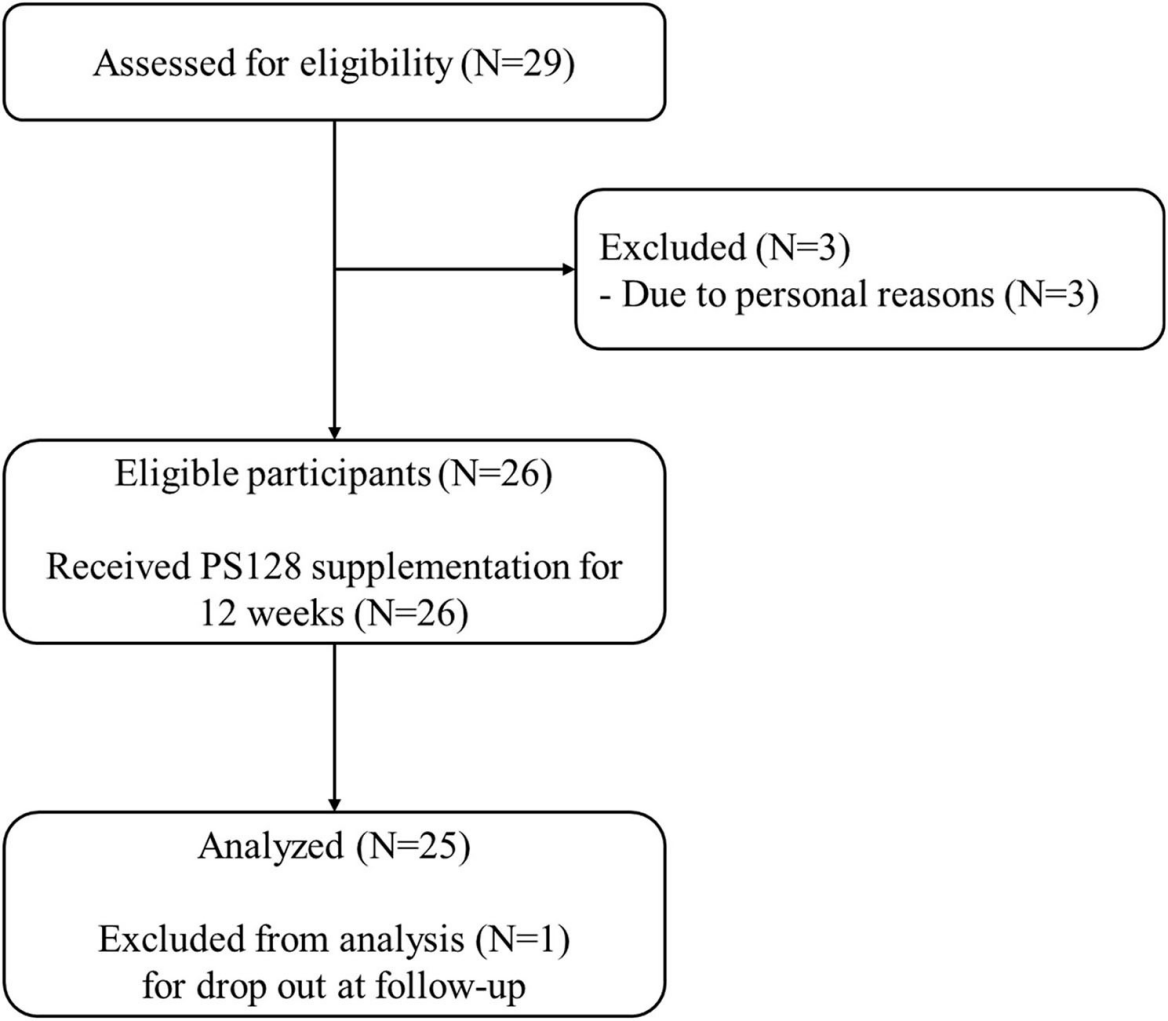
Study flow diagram.

The present study was conducted as an open-label, single-arm, baseline-controlled trial to evaluate the effects of PS128 supplementation on PD. The inclusion criteria were: (i) diagnosis of idiopathic PD, (ii) according to the record of ON/OFF diary for 3 consecutive days, the patient's daily OFF periods were more than 3 h a day, and (iii) age 40–80 years. The exclusion criteria were: (i) use of antibiotics within the preceding 1 month, (ii) use of other probiotic products (in the form of sachet, capsule, or tablet) within the preceding 2 weeks, (iii) previous surgery on the liver, bladder, or gastrointestinal tract, (iv) history of inflammatory bowel disease or cancer, (v) known allergy to probiotics, (vi) comorbid dementia (Mini-Mental State Examination [MMSE] score ≤ 26) or major depression (Beck Depression Inventory-II [BDI-II] score ≥ 29), (vii) history of deep brain stimulation, and (viii) ongoing artificial enteral or intravenous nutrition. In this study, 26 eligible subjects were enrolled, 25 completed the trial, and 1 dropped out due to personal reason (withdrew consent) (Figure 1). At the first screening visit, the principal investigator (PI) would arrange the interview with subjects to ensure whether he/she had experienced any differences in his/her symptoms between levodopa doses. For some patients, ON/OFF fluctuations were somewhat predictable but the symptoms experienced each time still varied. Once the PI confirmed that the subjects understood what ON/OFF meant and felt like, the PI would teach them how to fill out a symptom diary. Subjects had the chance to practice for 3 days, and once the diary was recorded correctly and properly, a new diary would be given to the subjects to record for 7 days. All patients were administered two capsules containing PS128 (30 billion colony-forming units per capsule) daily for 12 weeks. PS128 is a food supplement approved by the Taiwan Food and Drug Administration, and toxicological assessments have indicated that PS128 is safe for human supplementation (22). Compliance rate was assessed by counting the remaining capsules.

The study design followed the guidelines laid down in the Declaration of Helsinki and all procedures were conducted by trained testers at the Professor Lu Neurological Clinic, with approval by the Institutional Review Board of Antai Medical Care Cooperation, Antai Tian-Sheng Memorial Hospital (TSMH IRB No./Protocol No: 18-130-A). Written informed consent was obtained from all subjects.

### Assessment of Outcomes

In this study, the primary measurements were the Unified Parkinson's Disease Rating Scale part III (UPDRS-III) motor scores and changes in patient's “ON-OFF” diary recordings, and modified Hoehn and Yahr scale (mHYS). The secondary measurements were the 39-item Parkinson's Disease Questionnaire (PDQ-39), Non-Motor Symptoms Scale (NMSS), BDI-II, patient assessment of constipation symptoms (PAC-SYM), Patient Global Impression of Change (PGI-C), and other metabolic profiles. All clinical assessments were performed after 12 h of overnight withdrawal from antiparkinsonian medications (24 h of withdrawal was needed if the participants were taking prolonged release dopaminergic agonists). Therefore, UPDRS-III and mHYS were scored in the OFF state the next morning as well as the metabolic profiles. Then, the medications were self-administered by each patient before the secondary measurements. The UPDRS, mHYS, PDQ-39, NMSS, BDI-II, PAC-SYM, and PGI-C were scored 40–60 min later in the patients' best ON state. For safety assessment, physical and neurological examinations were performed to evaluate the overall health of the subjects at weeks 0 and 12. In addition, all subjects were actively monitored for the occurrence of adverse events by telephone at least once per week.

### Biochemical Measurements

A blood sample of 15 mL and a urine sample of 10 mL were collected at weeks 0 and 12. The serum high-sensitivity C-reactive protein level was determined by turbidimetric immunoassay (23). The plasma myeloperoxidase (MPO) and urinary 8-hydroxy-2'-deoxyguanosine were measured by enzyme-linked immunosorbent assay. The plasma glutathione peroxidase and total antioxidant capacity were measured by Ransel test kits (Randox Laboratories Ltd., UK) and ferric-reducing ability (24), respectively. The urinary creatinine (CRE) level was determined by MeDiPro creatinine test. All procedures were performed according to the manufacturer's instructions.

### Statistical Methods

All statistical analyses were analyzed using SPSS software (Version 22.0. Armonk, NY: IBM Corp.). Descriptive statistics were reported as the mean ± standard deviation (SD). The sign rank test was applied to the ordinal scale when a significant difference was detected. This test was applied to compare measures of total UPDRS, UPDRS-I, UPDRS-II, UPDRS-III, UPDRS-IV, tremor subscores, rigidity subscores, akinesia subscores, postural instability gait disorder (PIGD) subscores, mHYS, diary recordings, PDQ-39, BDI-II, and PAC-SYM between baseline (V0) and post-12 weeks (V1). The calculation of effect size is (Chohn's d) = mean of change/SD. The chi-square test applied to the nominal scale when a significant difference was detected of NMSS. The differences of metabolic parameters between baseline and probiotics intervention were determined by paired student *t*-test. The data were reported as mean ± SDs. Statistical significance was determined as *p* < 0.05.

## Results

### Baseline Characteristics

Table 1 presents the clinical and baseline demographic data of the patients. As shown in Figure 1, 25 eligible participants (eight female participants) completed the study. The age of subjects was 61.84 ± 5.74 years (range: 52–72 years), and the age at onset was 51.72 ± 6.59 years (range: 40–65 years). The duration of disease was 10.12 ± 2.3 years (range: 5–14 years). The MMSE score was 28.84 ± 1.68 (range: 25–30) and their levodopa equivalent daily dosage was 1063.4 ± 209.5 mg/daily (range: 675–1,560 mg/daily). All participants remained on the same dosage of anti-parkinsonian and other drugs throughout the study. All tested capsules were used by the patients during the intervention leading to a high compliance rate in this study.

**Table 1:** Demographic characteristics of the patients with PD.

**Variables**	**Mean (*N* = 25)**	**Range**
Gender	Female/Male = 8/17	–
Age, years	61.84 ± 5.74	52–72
Age at onset, years	51.72 ± 6.59	40–65
Duration of disease, years	10.12 ± 2.3	5–14
Height, cm	162.54 ± 7.29	147–184
Weight, kg	59.38 ± 7.78	47.5–79.5
Body mass index	22.49 ± 2.64	18.1–28.85
Education, years	12.86 ± 4.13	6–20
MMSE	28.84 ± 1.68	25–30
LEDD, mg/daily	1063.4 ± 209.5	675–1,560

*Mean ± SD; MMSE, Mini-Mental State Examination; LEDD, Levodopa equivalent daily dosage*.

### Primary Outcomes

As shown in Table 2, in the OFF state, administration of PS128 significantly decreased UPDRS motor scores (−3.08 ± 4.41, *p* = 0.004) and akinesia subscores (−1.96 ± 3.01, *p* = 0.012). In addition, a trend toward a greater reduction in rigidity subscores (−0.60 ± 1.35, *p* = 0.057) was observed after PS128 intervention. However, PS128 supplementation did not indicate any significant impact on tremor, rigidity, PIGD subscores, or mHYS. In the ON state, PS128 significantly reduced UPDRS motor scores (−2.56 ± 5.36, *p* = 0.007) and total UPDRS scores (−3.76 ± 6.04, *p* = 0.003), while the other scores did not improve. As shown in Table 3, PS128 intervention not only significantly reduced the duration of the OFF period (−0.80 ± 1.85, *p* = 0.04), but also significantly increased the duration of the ON period (0.84 ± 1.86, *p* = 0.031).

**Table 2:** The OFF- and ON-states of the unified Parkinson's disease rating scale of patients with PD at baseline and after 12 weeks of intervention.

**Outcomes**	**Baseline (V0)**	**Range**	**Post-12 weeks (V1)**	**Range**	***P***	**Change (V1-V0)**	**ES**
**OFF-state**						
UPDRS-III	27.16 ± 7.11 (26)	14–46	24.08 ± 8.2 (24)	10–42	0.004	−3.08 ± 4.41	0.699
Tremor subscores	2.2 ± 2.27 (2)	0–8	2.04 ± 2.01 (1)	0–7	1.000	−0.16 ± 1.21	0.132
Rigidity subscores	5.48 ± 3 (6)	1–10	4.88 ± 3.06 (5)	0–10	0.057	−0.60 ± 1.35	0.443
Akinesia subscores	11.64 ± 3.4 (11)	4–20	9.68 ± 4.03 (10)	3–17	0.012	−1.96 ± 3.01	0.652
PIGD subscores	1.88 ± 0.97 (2)	0–4	1.68 ± 1.14 (1)	0–4	0.267	−0.2 ± 0.71	0.283
mHYS	2.4 ± 0.38 (2.5)	2–3	2.3 ± 0.38 (2)	2–3	0.125	−0.1 ± 0.25	0.400
**ON-state**						
UPDRS-I	2.64 ± 1.75 (2)	0–7	2.48 ± 1.64 (2)	1–7	0.607	−0.16 ± 1.03	0.156
UPDRS-II	9.7 ± 4.27 (10)	2–16	8.8 ± 3.8 (10)	2–15	0.383	−0.92 ± 2.89	0.319
UPDRS-III	17.56 ± 6.92 (16)	8–33	15 ± 7.76 (15)	4–36	0.007	−2.56 ± 5.36	0.477
UPDRS-IV	3.08 ± 1.19 (3)	2–6	2.96 ± 1.02 (3)	2–6	1.000	−0.12 ± 1.39	0.086
Total UPDRS	33 ± 10.65 (30)	15–51	29.24 ± 10.26 (30)	12–53	0.003	−3.76 ± 6.04	0.623
Tremor subscores	1.08 ± 1.19 (1)	0–4	0.72 ± 0.84 (1)	0–3	0.118	−0.36 ± 0.99	0.362
Rigidity subscores	3.48 ± 2.28 (4)	0–8	3 ± 2.58 (3)	0–10	0.302	−0.48 ± 1.56	0.308
Akinesia subscores	8.68 ± 3.42 (8)	4–21	7.36 ± 3.44 (7)	1–14	0.078	−1.32 ± 3.15	0.420
PIGD subscores	0.6 ± 0.91 (0)	0–3	0.44 ± 0.92 (0)	0–3	0.453	−0.16 ± 0.62	0.256
mHYS	2.12 ± 0.3 (2)	2–3	2.1 ± 0.32 (2)	1.5–3	1.000	−0.02 ± 0.18	0.114

*Change in negative value indicates improvement. Mean ± SD (Medium); p: signed rank test, α = 0.05, 2-tailed; ES: 0.2 small effect, 0.5 medium effect, 0.8 large effect; Tremor subscores: items 20-21 (tremor at rest, action or postural tremor of hands); Rigidity subscores: item 22 (rigidity); Akinesia subscores: items 23–26 (finger taps, hand movements, rapid alternating movements of the hands, and leg agility); PIGD subscores: items 29–30 (gait, postural stability)*.

*ES, effect size; UPDRS, unified Parkinson's disease rating scale; mHYS, modified Hoehn and Yahr scale*.

**Table 3:** Diary recordings of ON and OFF durations in patients with PD at baseline and after 12 weeks of intervention.

**Hours/per day**	**Baseline (V0)**	**Range**	**Post-12 weeks (V1)**	**Range**	***p***	**Change (V1-V0)**	**ES**
OFF period	6.52 ± 1.66 (6.33)	3.8–11	5.72 ± 2.64 (5.67)	0–10.33	0.04	−0.80 ± 1.85	0.43
Moderate	1.08 ± 1.71 (0)	0–5.33	0.86 ± 1.50 (0)	0–4.83	0.754	−0.22 ± 1.29	0.17
Mild	5.43 ± 2.39 (5.33)	0.17–11	4.86 ± 2.80 (5.67)	0–10.33	0.09	−0.57 ± 1.47	0.39
ON period	9.92 ± 1.76 (10.33)	5.17–12.5	10.76 ± 2.29 (11)	4.67–15.5	0.031	0.84 ± 1.86	−0.45
Sleep	7.56 ± 1.73 (7.83)	4.67–10.83	7.51 ± 1.68 (7.17)	4.17–10.70	0.552	−0.04 ± 0.78	0.05

*Change in negative value indicates improvement: off period; change in positive value indicates improvement: on period*.

*Mean ± SD (Medium); p: Wilcoxon signed rank test, α = 0.05, 2-tailed; ES: 0.2 small effect, 0.5 medium effect, 0.8 large effect*.

*ES, effect size*.

### Secondary Outcomes

Comparing PDQ-39 values of patients with PD, the single index score significantly reduced after the 12-week intervention (−5.68 ± 8.55, *p* = 0.031) (Table 4), as well as mobility (−6.1 ± 12.73, *p* = 0.049), activities of daily living (−6.5 ± 11.85, *p* = 0.039), stigma (−8.75 ± 16.73, *p* = 0.039), and cognition (−6.75 ± 11.11, *p* = 0.021) dimensions. After 12 weeks of PS128 administration, the NMSS of none of the patients with PD improved significantly (Table 5). Most of the patients with PD expressed satisfaction with the PS128 intervention, and the PGI-C scores improved in 17 patients (68%) (Table 6). In addition, no adverse events related to PS128 intervention were found by the safety assessment.

**Table 4:** The 39-item Parkinson's disease questionnaire of patients with PD at baseline and after 12 weeks of intervention.

**PDQ-39**	**Baseline (V0)**	**Range**	**Post-12 weeks (V1)**	**Range**	***p***	**Change (V1-V0)**	**ES**
Single index	19.86 ± 9.6	3.39–40.42	14.18 ± 8.61	3.44–40.42	0.031	−5.68 ± 8.55	0.664
Mobility	19.2 ± 12.33	2.5–45	13.1 ± 13.43	0–62.5	0.049	−6.1 ± 12.73	0.479
Activities of daily living	17.83 ± 15.24	0–54.17	11.34 ± 8.8	0–29.17	0.039	−6.5 ± 11.85	0.548
Emotional well-being	19.67 ± 16.07	0–75	13.67 ± 16.65	0–75	0.077	−6 ± 10.96	0.547
Stigma	23.5 ± 17.33	0–75	14.75 ± 13.12	0–43.75	0.039	−8.75 ± 16.73	0.523
Social support	7.67 ± 7.95	0–25	6 ± 8.51	0–25	0.344	−1.67 ± 5.89	0.283
Cognitions	28 ± 17.96	0–62.5	21.25 ± 16.54	0–62.5	0.021	−6.75 ± 11.11	0.608
Communication	21 ± 16.69	0–58.33	15.33 ± 12.88	0–50	0.146	−5.67 ± 13.55	0.418
Bodily discomfort	22 ± 16.3	0–66.67	18 ± 12.43	0–41.67	1.000	−4 ± 15.98	0.250

*Change in negative value indicates improvement. Mean ± SD; p: signed rank test, α = 0.05, 2-tailed; ES: 0.2 small effect, 0.5 medium effect, 0.8 large effect*.

*PDQ-39, 39-item Parkinson's disease questionnaire; ES, effect size*.

**Table 5:** The non-motor symptoms of patients with PD at baseline and after 12 weeks of intervention.

**Outcomes**	**Baseline (V0)**		**Post-12 weeks (V1)**		***p***
Total NMSS	6.84 ± 4.65 (5)	Range 0–18	6.28 ± 4.35 (6)	Range 0–16	0.115
*NMS Domain, %*	No	Yes	No	Yes	*p'*
Gastrointestinal tract	24	76	20	80	0.625
Urinary tract	60	40	48	52	0.063
Sexual function	48	52	48	52	1
Cardiovascular	80	20	80	20	1
Cognitive function	44	56	44	56	1
Mood disorders	68	32	80	20	1
Sleep disorders	68	32	68	32	0.727
Impulse control disorders	100	0	100	0	NA
Others	48	52	48	52	1
BDI-II	8.6 ± 6.4 (7)	1–25	7.64 ± 7.08 (5)	0–34	1.000
PAC-SYM	7.8 ± 4.92 (8)	0–17	6.32 ± 5.1 (5)	0–20	0.108

*Mean ± SD (Medium); p', Chi square; NMSS, Non-Motor Symptoms Scale; BDI-II, Beck Depression Inventory-II; PAC-SYM, patient assessment of constipation symptoms*.

**Table 6:** The PS128 intervention improves patient global impression of change scores.

**PGI-C at V1 (post 12-weeks)**	**No**.	**%**	**Accumulative, %**
Very much improved	1	4	4
Much improved	4	16	20
Minimally improved	12	48	68
No change	7	28	96
Minimally worse	1	4	100
Much worse	0	0	100
Very much worse	0	0	100

*PGI-C, patient global impression of change*.

### Changes in Metabolic Parameters

**Figure 2:**
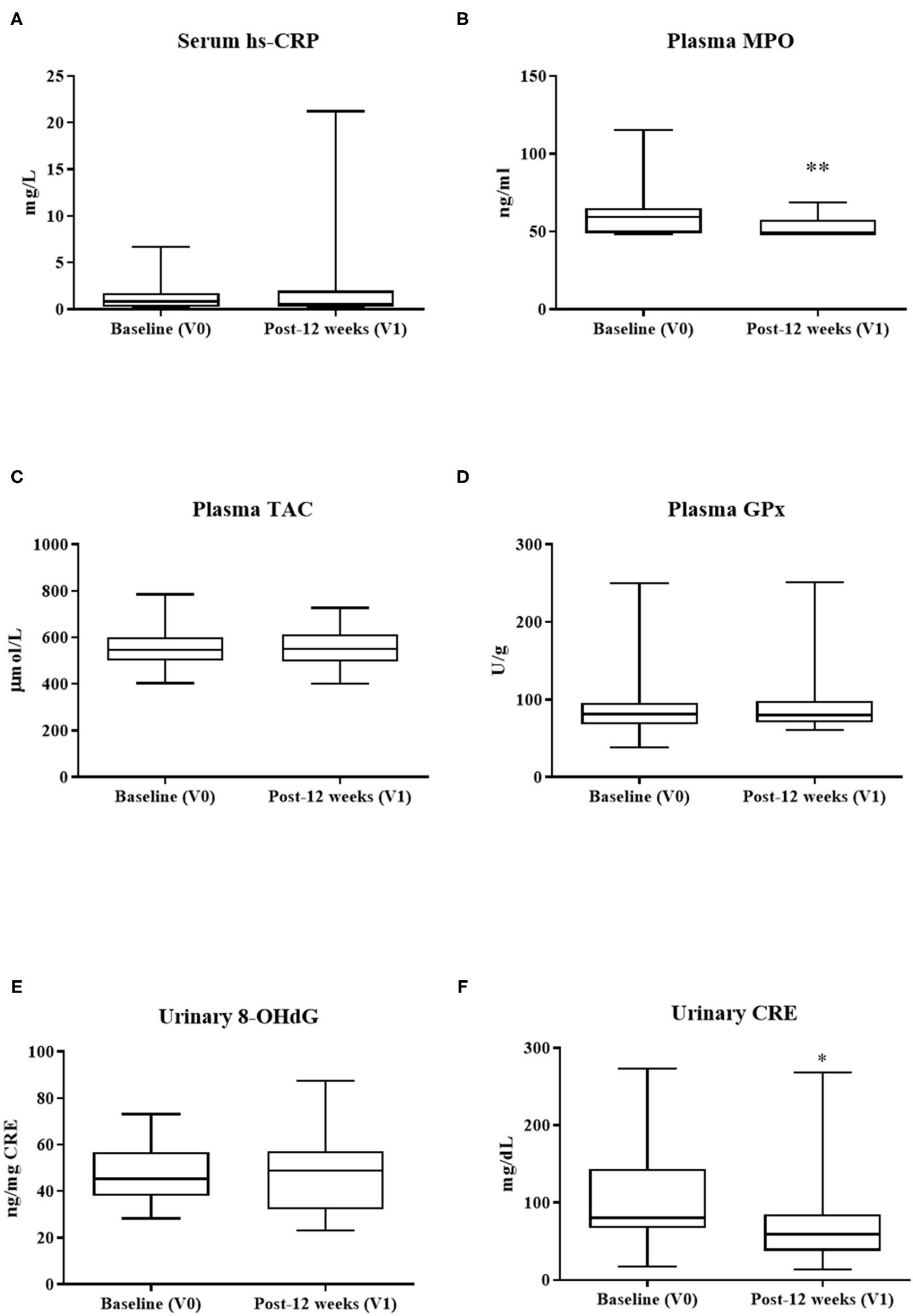
The effect of PS128 intervention on metabolic parameters of **(A)** hs-CRP, **(B)** MPO, **(C)** TAC, **(D)** GPx, **(E)** 8-OHdG, and **(F)** CRE. ^*^p < 0.05; ^**^p < 0.01 as compared to baseline (V0). Mean ± SD; *P*, Obtained from paired *t* test; hs-CRP, high-sensitivity C-reactive protein; MPO, myeloperoxidase; TAC, total antioxidant capacity; GPx, glutathione peroxidase; 8-OHdG, 8-hydroxy-2'-deoxyguanosine; CRE, creatinine.

Consumption of PS128 significantly reduced the level of plasma MPO (*p* < 0.01) (Figure 2). PS128 intervention also decreased the urinary CRE levels (*p* = 0.03) (Figure 2). There was no statistical difference in other metabolic parameters after PS128 intervention.

## Discussion

To our knowledge, this study is the first to report that a single strain probiotic can improve the UPDRS motor score of patients with PD in both the OFF and ON states. The UPDRS motor score decreased by 3.08 (11.3% improvement, 3.08/27.16) in the OFF state and 2.56 (14.6% improvement, 2.56/17.56) in the ON state (Table 2). Akinesia is related to dysfunction of automaticity and can affect almost all activities of daily life (25, 26). Thus, akinesia is one of most distressing motor disturbances experienced by patients with PD. In this study, the akinesia subscore decreased by 1.96 (16.8% improvement, 1.96/11.64) in the OFF state (Table 2). In agreement with the results from the akinesia subscore in the OFF state (Table 2), the activities of daily living dimension of the PDQ-39 (Table 4) was significantly improved by PS128 intervention. The PDQ-39 value suggested that the quality of life of patients with PD was less affected by disability. Moreover, the activities of daily living dimension of the PDQ-39 indicates the limitations in activities of daily living such as washing oneself, dressing oneself, and writing. In addition, the diary recording data suggested the add-on effect of PS128 in patients with PD by prolonging the ON period and decreasing the OFF period (Table 3). The above data suggested that PS128 intervention had add-on effects by improving the quality of life of patients with PD. The evidence for the effects of probiotics on ameliorating PD remains limited. The majority of probiotics research on patients with PD has been performed using a mixture of probiotics and have investigated the effect of gastrointestinal functions. It has been found that the consumption of a mixture of probiotics resulted in a reduced score on the UPDRS (27). Another clinical study reported that supplementation of a mixture of probiotics ameliorated inflammation in patients with PD (28). In addition, three studies demonstrated that treatment with a mixture of probiotics or fermented milk containing probiotics resulted in improved abdominal pain, bloating, and constipation, respectively (29–31).

MPO is a peroxidase enzyme that can produce reactive oxygen and nitrogen species (32). It has been reported that ablation of MPO can reduce neurodegeneration in PD-model mice (33). Recently, Makia et al. reported that MPO plays a crucial role in the aggregation of α-synuclein and deterioration of motor impairment (34). In addition, several studies have suggested that MPO inhibitors can be therapeutic in neurodegenerative diseases (35–38). It has been reported that PS128 intervention not only significantly improves motor impairment but can also restore the survival of dopaminergic neurons in the substantia nigra and striatum of PD-model mice (18). The immune system plays an important role in the pathophysiology of PD (39). In comparison with healthy subjects, hyperactivation of microglial cells and induction of microglia-derived cytokines were found in the patients with PD (40–42). Notably, administration of PS128 ameliorated neuroinflammation, increased antioxidant activity, and enhanced levels of neurotrophic factor in PD-model mice (18).

More than 30% of patients with PD suffer from gastrointestinal symptoms (43). Gastrointestinal problems such as constipation and delayed gastric emptying are frequently observed in patients with PD (44). However, we did not observe a significant improvement in NMSS and PAC-SYM in patients with PD after a 12-week intervention with PS128 (Table 5). It is possible that all subjects remained on their gastrointestinal-related drugs, including magnesium oxide, bisacodyl, and sennoside A+B calcium, throughout the study (Supplementary Table 1). The effect of PS128 on gastrointestinal symptoms requires further study.

Emerging evidence shows that misfolded α-synuclein seems to appear in the EECs and enteric nerves first, and then in the brain (45–47). Experimentally, α-synuclein can be spread from the gut to brain via the vagus nerve, resulting in the loss of dopaminergic neurons in the substantia nigra (6, 48). Besides, a previous study has reported that oral administration of PS128 to rats increased the level of peripheral serotonin produced by EECs (16, 17), suggesting that there is an interaction between PS128 and EECs. Therefore, it is possible that PS128 regulates the production of α-synuclein in the EECs and enteric nerves, thereby alleviating the progression of PD, and the possibility remains to be studied.

There are a few limitations to the present study. Firstly, this study is an open-label trial rather than placebo-controlled, blinded, or randomized study. Therefore, follow-up large-scale, randomized controlled studies are needed to determine the efficacy of PS128 in patients with PD at different stages. Secondly, the effect of PS128 on the gut microbiome was not evaluated in this pilot study. Emerging evidence has demonstrated that the gut microbiome plays an important role in development and maintenance of human physiology (1, 49, 50). Recently, alteration of the gut microbiome composition in patients with PD has been found (51–53). These changes are considered to induce the loss of dopaminergic neurons by triggering inflammation, reduce levels of neuroprotective factors, and produce neurotoxins into the systemic circulation (1). However, whether a specific microbial species regulates the pathophysiology of PD remains a controversial issue. Whether PS128 affects the symptoms of PD via modulation of the gut microbiome warrants further investigation.

## Conclusion

In summary, the present study demonstrated that supplementation with PS128 for 12 weeks significantly improved the UPDRS motor scores in both the OFF and ON states, the duration of the ON period, and the quality of life of patients with PD. This pilot study suggests that PS128 could be considered as a therapeutic adjuvant for the treatment of PD. Further studies are required to determine the role of PS128 on inflammatory/metabolic parameters and the gut microbiome in regulating PD.

## Data Availability Statement

The original contributions presented in the study are included in the article/Supplementary Material, further inquiries can be directed to the corresponding authors.

## Ethics Statement

The studies involving human participants were reviewed and approved by Institutional Review Board of Antai Medical Care Cooperation, Antai Tian-Sheng Memorial Hospital (TSMH IRB No./Protocol No: 18-130-A). The patients/participants provided their written informed consent to participate in this study.

## Author Contributions

H-CC and Y-SK designed the study, performed all the clinical assessments, analyzed, and interpreted data. C-CC and Y-HW recruited the patients and edited the manuscript. H-CC wrote the manuscript. Y-CT critically reviewed the manuscript but was not involved in any data collection or analysis. C-SL supervised the study and critically edited the manuscript. All authors listed have contributed substantially to the work as noted and agreed to submit the manuscript.

## Conflict of Interest

Y-CT owns stock of Bened Biomedical Co., Ltd. The remaining authors declare that the research was conducted in the absence of any commercial or financial relationships that could be construed as a potential conflict of interest.
