# Supplementary material for: The Add-On Effect of Lactobacillus plantarum PS128 in Patients With Parkinson's Disease: A Pilot Study
Source: Front Nutr. 2021 Jun 30;8:650053. doi: 10.3389/fnut.2021.650053 (PMC8277995; doi:10.3389/fnut.2021.650053)
Supplement: Supplementary file 1 [file Table_1.docx]

**Supplementary Table 1. Medications of patients with PD.**

| **Medications, mg/daily** | **Screening subject, N = 29** | | |  | **Completed, N = 25** | | |
| --- | --- | --- | --- | --- | --- | --- | --- |
|  | n (%) | Mean ± SD | Range |  | n (%) | Mean ± SD | Range |
| LEDD^*^ | 29 (100) | 1044.66 ± 214.17 | 625–1560 |  | 25 (100) | 1063.4 ± 209.5 | 675–1560 |
| Propranolol^*^ | 24 (83) | 39.79 ± 14.93 | 15–80 |  | 20 (80) | 190 ± 369.97 | 0–1500 |
| Magnesium Oxide | 9 (31) | 611.11 ± 416.67 | 250–1500 |  | 8 (32) | 571.43 ± 472.46 | 250–1500 |
| Bisacodyl | 2 (7) | 12.5 ± 3.54 | 10–15 |  | 2 (8) | 1 ± 3.54 | 0–15 |
| Sennoside A+B calcium | 4 (14) | 35 ± 10 | 20–40 |  | 3 (12) | 4 ± 11.55 | 0–40 |
| Clonazepam | 27 (93) | 1.18 ± 0.75 | 0.5–3 |  | 23 (92) | 1.12 ± 0.81 | 0–3 |
| Quetiapine | 3 (10) | 25 ± 0 | 25–25 |  | 3 (12) | 3 ± 8.29 | 0–25 |

LEDD: Levodopa equivalent daily dosage.

^*^ 12 or 24 hours of withdrawal before the assessment.
